# Efficacy of probiotic *Streptococcus thermophilus* in counteracting TGF-β1-induced fibrotic response in normal human dermal fibroblasts

**DOI:** 10.1186/s12950-022-00324-9

**Published:** 2022-12-19

**Authors:** Francesca Lombardi, Francesca Rosaria Augello, Serena Artone, Blerina Bahiti, Jenna Marie Sheldon, Maurizio Giuliani, Maria Grazia Cifone, Paola Palumbo, Benedetta Cinque

**Affiliations:** 1 Department of Life, Health and Environmental Sciences, University of L’Aquila, Via Pompeo Spennati, Building “Rita Levi Montalcini” (Delta 6), 67100 L’Aquila, Italy; 2 Dr. Kiran C Patel College of Osteopathic Medicine, Nova Southeastern University, Fort Lauderdale, FL USA

**Keywords:** Skin fibrosis, TGF-β1, *S. thermophilus*, Fibrotic markers, Smad signaling, β-catenin, PPARγ

## Abstract

**Background:**

Abnormal and deregulated skin wound healing associated with prolonged inflammation may result in dermal fibrosis. Since the current therapeutic strategies revealed unsatisfactory, the investigation of alternative approaches such as those based on the use of specific probiotic strains could provide promising therapeutic options. In this study, we aimed to evaluate whether the lysate from *S. thermophilus* could antagonize the fibrogenic effects of TGF-β1 in normal human dermal fibroblasts (NHDF).

**Methods:**

NHDF were exposed to TGF-β1 to establish a fibrotic phenotype. Proliferation rate and cell number were measured using the IncuCyte® Live Cell Imager system and the trypan blue dye exclusion test. Phenoconversion markers (α-SMA and fibronectin) and collagen I levels were assessed by western blot and immunofluorescence. The mRNA levels of TGF-β1 were evaluated by RT-PCR. The Smad2/3 phosphorylation level as well as β-catenin and PPARγ expression, were assessed by western blot. The cell contractility function and migration of NHDF were studied using collagen gel retraction assay, and scratch wound healing assay, respectively. The effects of *S. thermophilus* lysate, alone or combined with TGF-β1, were evaluated on all of the above-listed parameters and markers associated with TGF-β1-induced fibrotic phenotype.

**Results:**

Exposure to the *S. thermophilus* lysate significantly reduced the key mediators and events involved in the abnormal activation of myofibroblasts by TGF-β1 within the fibrotic profile. The *S. thermophilus* treatment significantly reduced cell proliferation, migration, and myo-differentiation. In addition, the treatment with probiotic lysate reduced the α-SMA, fibronectin, collagen-I expression levels, and affected the collagen contraction ability of activated dermal fibroblasts. Moreover, the probiotic targeted the TGF-β1 signaling, reducing Smad2/3 activation, TGF-β1 mRNA level, and β-catenin expression through the upregulation of PPARγ.

**Conclusion:**

This is the first report showing that *S. thermophilus* lysate had a remarkable anti-fibrotic effect in TGF-β1-activated NHDF by inhibiting Smad signaling. Notably, the probiotic was able to reduce β-catenin and increase PPARγ levels. The findings support our point that *S. thermophilus* may help prevent or treat hypertrophic scarring and keloids.

**Supplementary Information:**

The online version contains supplementary material available at 10.1186/s12950-022-00324-9.

## Introduction

Pathological scarring of wounds, keloids, burn injuries, surgery traumas, or excessive and prolonged inflammation can cause cutaneous fibrosis. Skin fibrotic disorders are characterized by dysregulated deposition of extracellular matrix in the dermis, leading to skin function and architecture loss.

Although different inflammatory cytokines and growth factors are involved in skin fibrosis, the TGF-β1-activated pathway is currently considered to play a pivotal role in the fibrotic phenotype observed for fibroblasts isolated from keloids or hypertrophic scarring. In this context, the modulation of the TGF-β1 signaling in fibroblasts may confer a hopeful opportunity for the treatment of skin fibrosis.

TGF-β1 stimulates the activation and proliferation of fibroblasts, the primary cell type in the skin responsible for maintaining skin homeostasis. It also promotes the synthesis and deposition of extracellular matrix (ECM) proteins, inhibiting their degradation. During the pathological and abnormal skin repair process, the TGF-β1-activated fibroblasts acquire a highly contractile phenotype, significantly contributing to dermal fibrosis by excessive ECM deposition. Smad signaling, strongly implicated in skin fibrosis and activated by TGF-β1 that promotes the Smad 2/3 phosphorylation (canonical TGF-β pathway), enhances the expression of fibrogenic genes, such as *collagen I* (coll I) and *α-SMA* in activated fibroblasts [1]. Moreover, TGF-β1, upregulated in skin fibrosis, requires *de novo* synthesis by many cell types [2], and also through the autocrine induction of its gene expression in a positive feedback loop [3–5]. Another critical pathway in skin fibrosis is the Wnt/β-catenin signaling pathway [6]. β-catenin accumulation and its subsequent nuclear translocation may increase proliferation, migration, *collagen I* gene expression [7, 8], and the excessive deposition of ECM proteins [9], contributing to dermal fibrosis. An important crosstalk has been highlighted among Wnt-signaling, TGF-β1/Smad pathway and peroxisome proliferator activated receptor gamma (PPARγ) and it is well documented that Wnt/β-catenin and TGF-β1 signaling stimulate and coordinate with each other, in the process of fibrosis [9–11]. Notably, the β-catenin pathway is an important antagonist system of PPARγ signaling in several conditions [11], including fibrosis, and TGF-β1 acts on both these antagonist pathways, activating Wnt/β-catenin signaling and reducing PPARγ levels, which are significantly lowered in fibrotic skin compared to healthy skin [12]. PPARγ, a crucial transcription factor activated by natural or synthetic ligands, stimulates several target genes involved in the metabolism, immune response, and inflammation [13]. The activation of PPARγ leads to suppression of TGF-β1-stimulated myofibroblast differentiation and collagen synthesis in skin fibroblasts [14], suggesting the potentiality of PPARγ agonists to prevent skin fibrosis and excessive scarring [15–18].

To date, the available anti-scarring approaches including surgery, vascular laser therapy, and pressure therapy have limited efficacy on the final scar outcome. Therefore, there is a crucial need for more efficacious treatments for fibrotic disorders.

Currently, anti-fibrotic therapies are developed to target TGF-β1 signaling and modulate profibrotic mediators [19]. Although potentially powerful, such therapeutic approaches are usually expensive and not free from adverse events [20].

The potential of specific probiotic strains as topical therapeutic agents has been reported by previous studies on pathophysiological skin alterations, including wound infection and repair, acne, atopic dermatitis, psoriasis, seborrheic dermatitis, photoaging and skin aging [21–24].

The topical probiotic application could promote a positive balance in favor of beneficial bacteria eliminating pathogens, especially in those pathologies associated with skin dysbiosis, which is responsible for immune dysfunction and disrupting the skin barrier. The effects of improving skin health by topical probiotics are considered mainly based on three elements: antagonize pathogens, improve immunotolerance, induce anti-inflammatory effect, release bioactive molecules such as bacteriocins, modulins, antimicrobial peptides and propionic acid, able to inhibit the growth of pathogens, and recover skin barrier function [21]. Although the precise mechanisms by which probiotics improve skin health are not yet known, literature and patents related to the application of topical probiotics for the treatment of various skin disorders continue to grow [21, 23–26].

Recently, our group reported the first experimental evidence relative to the ability of the Vivomixx® high-concentration, multi-strain probiotic formulation to inhibit the TGF-β1-induced fibrotic phenotype in colonic intestinal fibroblast cells CCD-18Co. The exposure of TGF-β1-activated CCD-18Co to this probiotic formulation has been proven to reduce the collagen-I and α-SMA expression by interfering with Smad2/3 signaling and counteracting TGF-β1 neosynthesis [27]. The beneficial anti-fibrotic effect of the probiotic formulation on the intestinal epithelial model led us to investigate whether one of its components, the *S. thermophilus* DSM 24731 strain, could also influence dermal fibrotic mechanisms. This choice was supported by our previous in vivo studies evidencing the beneficial anti-inflammatory and anti-aging effects of the *S. thermophilus* DSM 24731. In particular, a significant increase in skin ceramide levels was registered by our group in healthy subjects after treatment in vivo with a cream containing *S. thermophilus* lysate [28]. The presence of high levels of neutral sphingomyelinase activity in this probiotic was responsible for the observed increase of stratum corneum ceramide levels, thus leading to an improvement in barrier function and maintenance of stratum corneum flexibility. The observed effects could be attributed the presence of high levels of neutral sphingomyelinase activity in *S. thermophilus*, an enzyme responsible for the observed increase of stratum corneum ceramide levels, thus leading to an improvement in barrier function and maintenance of stratum corneum flexibility. Our group also reported that topical administration of *S. thermophilus* in patients with atopic dermatitis led to a significant improvement in erythema, scaling, and pruritus [29]. In another study we showed that the topical treatment of a *S. thermophilus*-containing cream on healthy elderly women also led to significant and relevant increase of stratum corneum ceramide levels and decrease of transepidermal water loss and capacitance of the treated skin [30]. We also depicted the use of topical *S. thermophilus* in slowing the process of photoaging, reducing oxidative stress and improving the skin barrier function [31]. Stimulated by these previous results, we focused on investigating the anti-fibrotic properties of *S. thermophilus* lysate on TGF-β1-activated normal human dermal fibroblasts (NHDF) and its possible use as an effective option in managing skin fibrotic disorders.

## Results

### Effect of *S. thermophilus* lysate on TGF-β1-induced cell proliferation and migration of NHDF

**Fig. 1:**
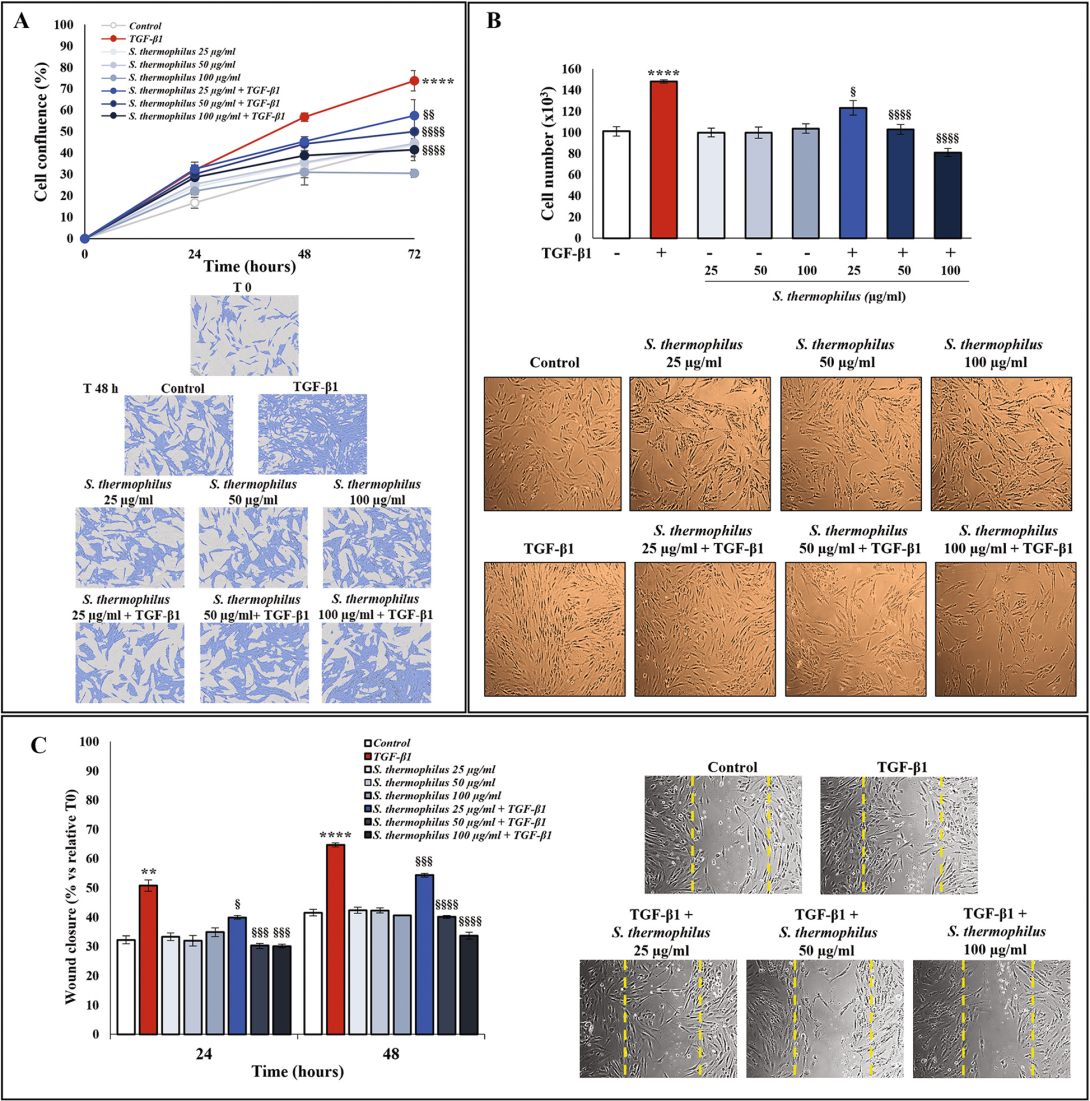
Effect of *S. thermophilus* lysate on TGF-β1-induced proliferation and migration of NHDF.** A** Growth curves of NHDF were analyzed as cell confluence percentage through IncuCyte® Live Cell Imager and monitored dynamically from 0 to 72 h. NHDF were starved for 24 h and then incubated until 3 days with TGF-β1 (10 ng/ml) in the presence or absence of *S. thermophilus* lysate (25, 50 or 100 µg/ml), in 0.5% FBS medium. Results relating to one representative out of three experiments performed in triplicate, are expressed as mean ± SD. **B** NHDF were treated for 48 h as reported in A and cell number was counted by trypan blue staining. The results, derived from three experiments performed in duplicate, are expressed as mean ± SEM. **C** Quantitative analysis of wound healing assay in NHDF treated as above in A. The wound closure was captured at 0, 24, and 48 h after scratch generation and expressed as the wound closure rate (% vs. relative T0) of scratched monolayers. Values are expressed as mean ± SEM of two independent experiments in duplicate. In each case, the comparative analysis of groups of data has been performed by the two-way analysis of variance (ANOVA) followed by post hoc Tukey’s test (** *P* < 0.01, **** *P* < 0.0001 vs. CNTR; § *P* < 0.05, §§ *P* < 0.01, §§§ *P* < 0.001, §§§§ *P* < 0.0001 vs. TGF-β1). Representative images of cell confluence, cell number and NHDF monolayer re-epithelialization (the yellow dashed lines indicate wound edges at T0) are inserted in A, B and C respectively

Cell proliferation was dynamically monitored every 6 h over a 72 h time period in NHDF monolayers, previously incubated with 0.5% FBS supplemented medium for 24 h and then stimulated with TGF-β1 at 10 ng/ml, alone or in presence of *S. thermophilus* lysate at 25, 50, or 100 µg protein/ml. As showed in Fig. 1A, the TGF-β1-induced activation of NHDF was confirmed. The treatment with TGF-β1 at 10 ng/ml also enhanced the proliferation rate expressed as cell confluence percentage by day 1, producing an effect that increased over the 72 h (~ 50% more than the untreated control cells). When the cells were exposed to *S. thermophilus* lysate plus TGF-β1, the percentage of cell confluence significantly decreased in time- and concentration-dependent manners with respect the TGF-β1 alone assuming values comparable to those determined for basal levels. Such findings provide evidence that bacterial lysate was able to inhibit TGF-β1-induced proliferation (Fig. 1A). Differently, the bacterial lysate alone did not significantly affect the cell proliferation rate, which remained similar to the basal level in control cells.

Evaluation of the impact of *S. thermophilus* lysate on cell number and viability gave similar results. After the treatment with different concentrations of bacterial lysate, no change has been observed in the cell number of the control group, while the addition of *S. thermophilus* significantly inhibited the increase in the number of NHDF induced by TGF-β1 at 48 h (Fig. 1B). Notably, the observed effect was more evident by exposing cells to 50 and 100 µg/ml of bacterial lysate. The microscopic images of the cell density under the different treatment conditions confirmed the reduction in cell number contemporary highlighting that both the parallel oriented spindle-like shape, as well as the evident increase of cell density in the presence of TGF-β1, were clearly affected by co-treatment with bacterial lysate (Fig. 1B). The quantitative in vitro analysis of wound-closure rate showed that the closure percentage at 24 and 48 h increased in TGF-β1-activated NHDF compared with the control, while it decreased in a dose-dependent manner after the co-treatment with *S. thermophilus* lysate (Fig. 1C). Representative microscopy images taken at 48 h are also shown (Fig. 1C).

### Effects of *S. thermophilus* lysate on TGF-β1-induced myofibroblast phenoconversion

**Fig. 2:**
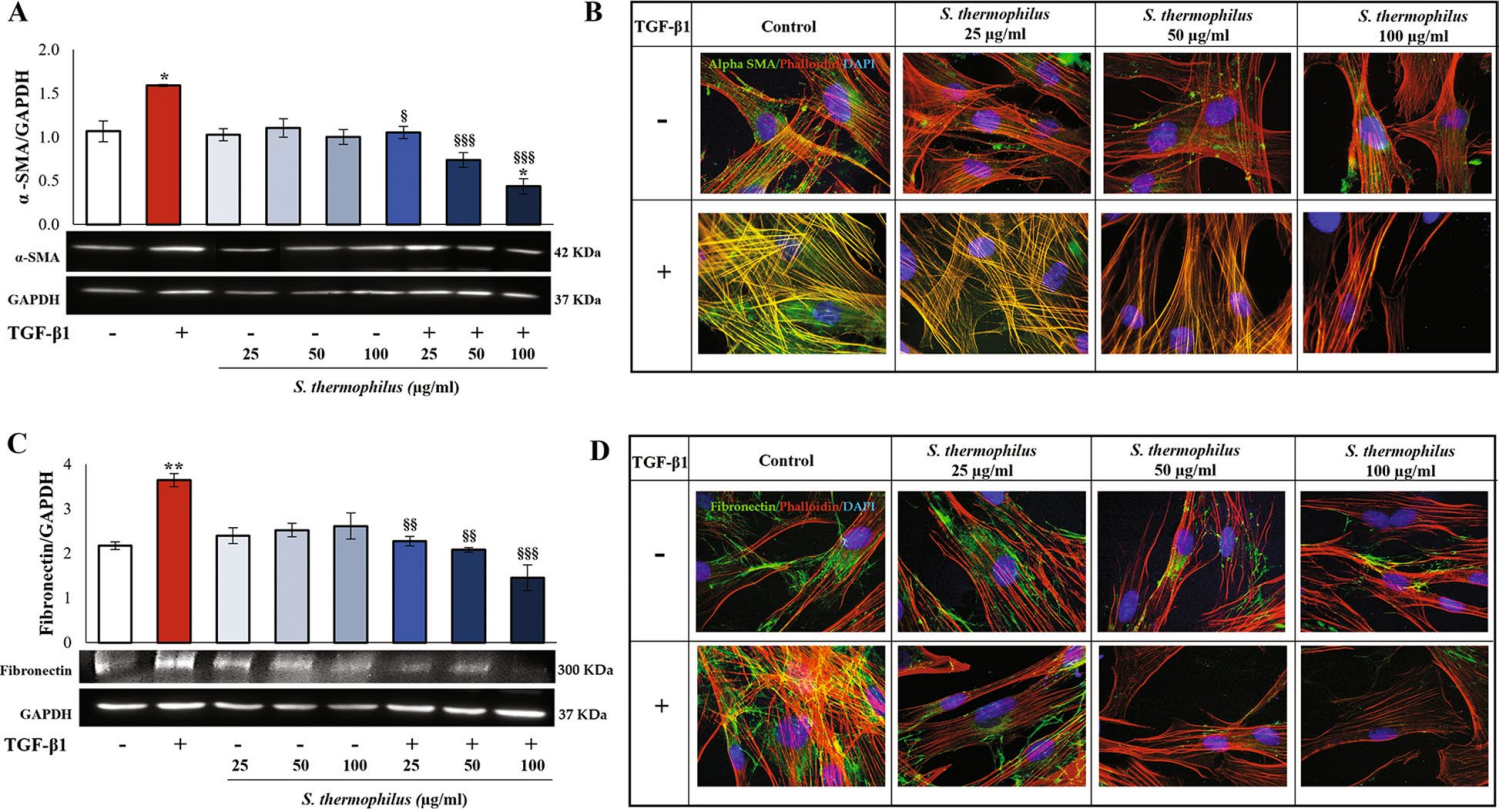
Effects of *S. thermophilus* lysate on TGF-β1-induced myofibroblast phenoconversion. Immunoblotting assays for **A** α-SMA and **C** fibronectin were performed on NHDF treated as previously reported. Densitometric analysis was performed by normalizing vs. GAPDH. Values are expressed as mean ± SEM of three independent experiments. For comparative analysis of data, a one-way analysis of variance (ANOVA) with post hoc Tukey’s test was used. (* *P* < 0.05, ** *P* < 0.01 vs. CNTR; § *P* < 0.05, §§ *P* < 0.01, §§§ *P* < 0.001 vs. TGF-β1). Representative images of each immunoblotting are shown. Representative immunofluorescence images of NHDF stained with anti-α-SMA antibody **(B)** (green) or anti-fibronectin antibody **(D)** (green) and with TRITC-phalloidin (red) to reveal F-actin. Nuclei were counterstained with DAPI (blue) (magnification 100 x). The images are representative of three independent experiments in duplicate

The degree of skin fibrosis can be determined by the expression of typical markers of phenoconversion of fibroblasts into myofibroblasts such as α-SMA and fibronectin and by cytoskeleton re-organization [2]. Thus, the effect of *S. thermophilus* lysate was evaluated on the α-SMA expression in TGF-β1-activated NHDF. Western blot analysis indicated that α-SMA protein levels were significantly up-regulated compared with the control cells (Fig. 2A). The addition of the bacterial lysate is associated to significant marked dose-dependent decrease of α-SMA expression in NHDF exposed to TGF-β1 for 48 h. The immunofluorescence analysis confirmed that in response to TGF-β1 activation, the network of actin-myosin bundles stained by phalloidin (red fluorescence) was highly decorated with α-SMA, as observed by intense and overlapped yellow/orange fluorescence. On the other hand, the addition of the bacterial lysate caused a weaker fluorescent signal relative to α-SMA, restraining the myofibroblast phenoconversion by TGF-β1 (Fig. 2B).

Immunoblotting and immunofluorescence analysis results showed that fibronectin protein expression followed a pattern similar to that of α-SMA characterized by a significant increase in TGF-β1-treated NHDF compared to control. The exposure to *S. thermophilus* lysate was associated to a dose-dependent significant decrease in the stimulatory effect of TGF-β1 also on fibronectin expression concerning either protein quantity or determined positive cells (Fig. 2C and D). No effects on α-SMA and fibronectin expression were associated with the exposure to the *S. thermophilus* lysate alone.

### Ability of *S. thermophilus* lysate to affect collagen I production in the TGF-β1-actived NHDF and ECM remodeling

**Fig. 3:**
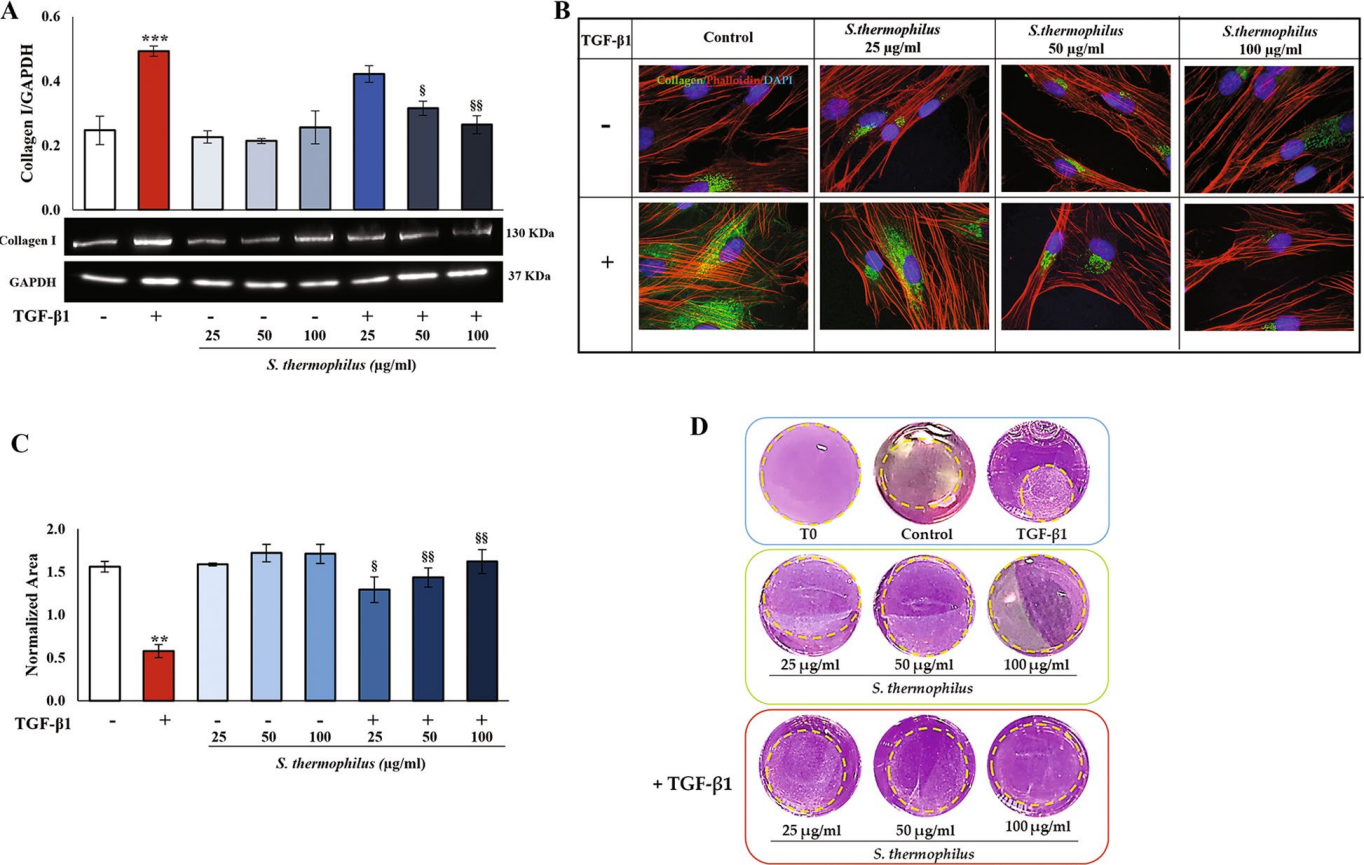
Effects of *S. thermophilus* lysate on collagen I production in TGF-β1-activated NHDF and ECM remodeling.** A** Immunoblotting assay for collagen I was performed on NHDF treated as previously described. Following densitometric analysis, obtained values were normalized vs. GAPDH. Values are expressed as mean ± SEM of three independent experiments performed in duplicate. Images from one representative out of three independent experiments are presented. **B** Representative immunofluorescence images of NHDF stained with anti-collagen I antibody (green) and with TRITC-phalloidin (red) to reveal F-actin. Nuclei were counterstained with DAPI (blue) (magnification 100 x). The images are representative of three independent experiments in duplicate. **C** Collagen gel retraction assay of NHDF-populated collagen lattices. The gel contraction was digitally photo-documented, and the gel area was measured with ImageJ and normalized to pre-release area (T0). Values of normalized area are expressed as mean ± SEM of two independent experiments in duplicate. **D** Representative images of collagen gel pre-release (T0) and taken 48 h after treatments are shown. In all cases, the comparative analysis of data has been carried out by using one-way analysis of variance (ANOVA) followed by post hoc Tukey’s test (** *P* < 0.01 vs. CNTR; *** *P* < 0.001 vs. CNTR; § *P* < 0.05, §§ *P* < 0.01 vs. TGF-β1)

We next studied if the *S. thermophilus* lysate affected the ability of TGF-β1-activated NHDF to produce collagen I, as a mark of extracellular matrix deposition. As shown in Fig. 3A, western blot analysis revealed that NHFD exposed to TGF-β1 (10 ng/ml) for 48 h and showing a change in their phenotype towards activated myofibroblasts, expressed higher levels of Coll I when compared to the control. Conversely, the exposure to *S. thermophilus* lysate (50 and 100 µg/ml) for 48 h led to a marked decrease of the Coll I level in TGF-β1-activated NHDF. Immunofluorescence analysis confirmed these results (Fig. 3B). TGF-β1-treated cells appeared intense with Coll I stain, while the *S. thermophilus* lysate at the two highest concentrations induced a substantial reduction of staining for Coll I, to the extent where the relative fluorescent signals appeared to be weak and diffuse similarly to the untreated cells. The lower 25 µg/ml concentration of bacterial lysate slightly reduced the TGF-β1-induced effect at 48 h.

To investigate the functional consequences of *S. thermophilus* lysate exposure on the contractile machinery of NHDF, a 3D collagen gel contraction assay consisting of cell-collagen I matrices was used to mimic the exertion of traction forces representing a critical feature of activated myofibroblasts. Obtained results showed that TGF-β1 stimulation for 48 h led to a marked decrease in the size of the collagen lattices, as a sign of collagen gel shrinkage, compared with the control group, indicating the increased contractility of fibroblasts embedded in collagen lattices (Fig. 3C and D). Of note, in presence of *S. thermophilus* treatment, the decrease in the collagen lattice size induced by TGF-β1 was hindered in a concentration-dependent manner, thus supporting the effect of the bacterial lysate on the collagen contraction ability of activated dermal fibroblasts. No statistically significant differences were observed between the retraction profiles determined for controls and NHDF treated with *S. thermophilus* alone. Similarly, their diameters did not decrease respect to the T0 considering a similar area (Fig. 3D).

### Effects of *S. thermophilus* lysate on TGF-β1-induced Smad activation

**Fig. 4:**
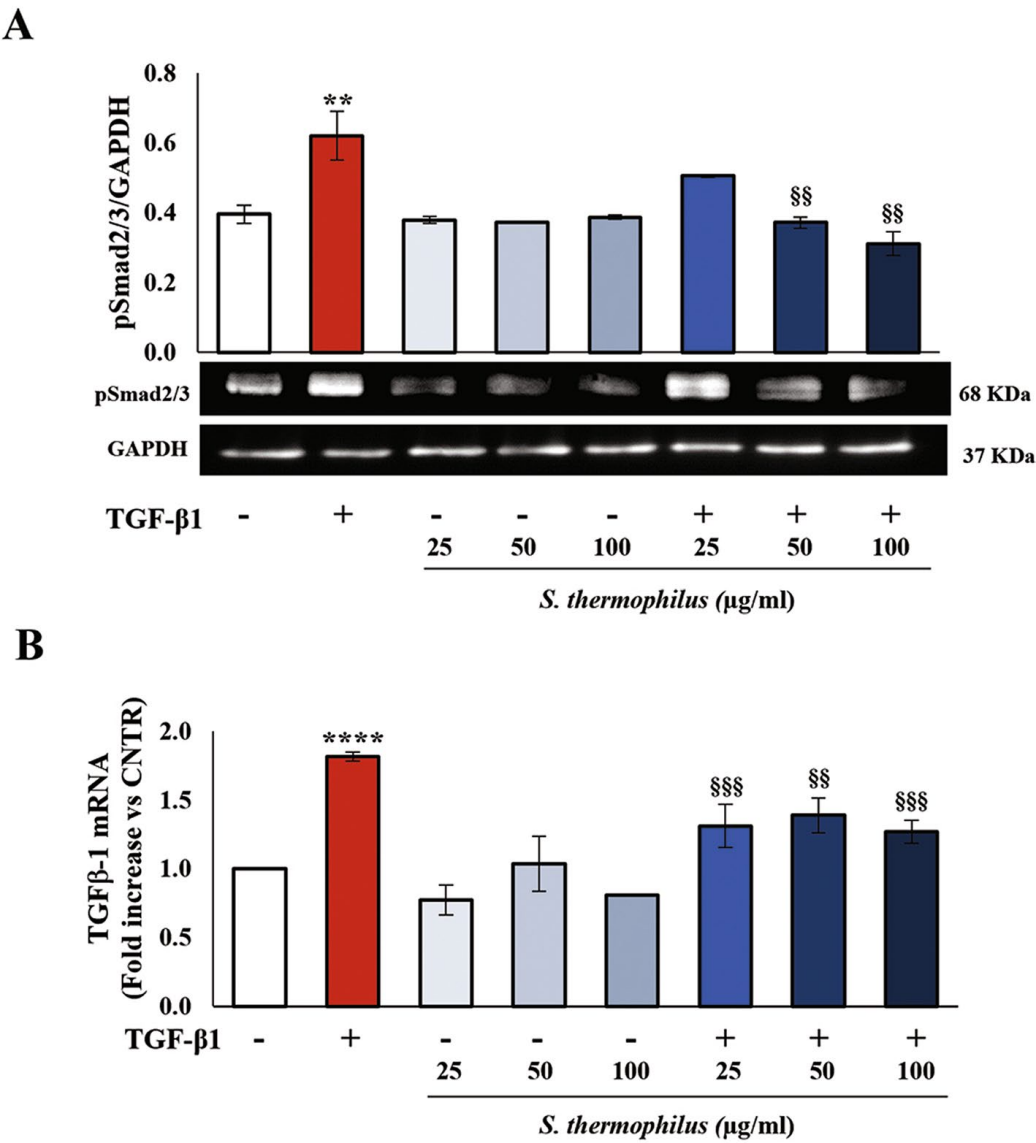
Effects of *S. thermophilus* lysate on TGF-β1-induced Smad signaling.** A** Immunoblotting assay for pSmad2/3 protein expression was performed on NHDF treated as described above. Following densitometric analysis, obtained values were normalized vs. GAPDH. Values are expressed as mean ± SEM of two independent experiments performed in duplicate. Representative images of immunoblotting for pSmad2/3 and GAPDH are shown. **B** The SYBRGreen Real-Time PCR analysis of the TGF-β1 gene was performed on NHDF. mRNA levels were relative to the amount of GAPDH mRNA. Data from one of two independent experiments in duplicate are shown as mean ± SD. In all cases, the comparative analysis of data has been carried out by using one-way analysis of variance (ANOVA) followed by post hoc Tukey’s test (** *P* < 0.01 vs. CNTR; **** *P* < 0.0001 vs. CNTR; §§ *P* < 0.01 vs. TGF-β1, §§§ *P* < 0.001 vs. TGF-β1)

In the canonical pathway, TGF-β1 acts through Smad signaling responsible for controlling collagen deposition and fibrogenesis [32]. As expected, the treatment of NHDF with TGF-β1 induced phosphorylation of Smad2/3 at 48 h (Fig. 4A). The simultaneous addition of probiotic prevented the TGF-β1-induced activation, thus keeping Smad2/3 phosphorylation levels in line with those of the untreated control.

Moreover, TGF-β1/Smad signaling pathway contributed to fibrotic phenotype promoting the induction of TGF-β1 gene transcription, as shown by qRT-PCR analysis, confirming previous studies [3–5]. Of note, the significant increase of TGF-β1 mRNA expression seen in activated-NHDF was strongly reduced by co-treatment with bacterial lysate for 48 h at all used concentrations (Fig. 4B). No effect on TGF-β1 gene expression was observed when the cells were treated with *S. thermophilus* lysate alone.

### Effect of *S. thermophilus* lysate on β-catenin and PPARγ expression in TGF-β1-activated NHDF

**Fig. 5:**
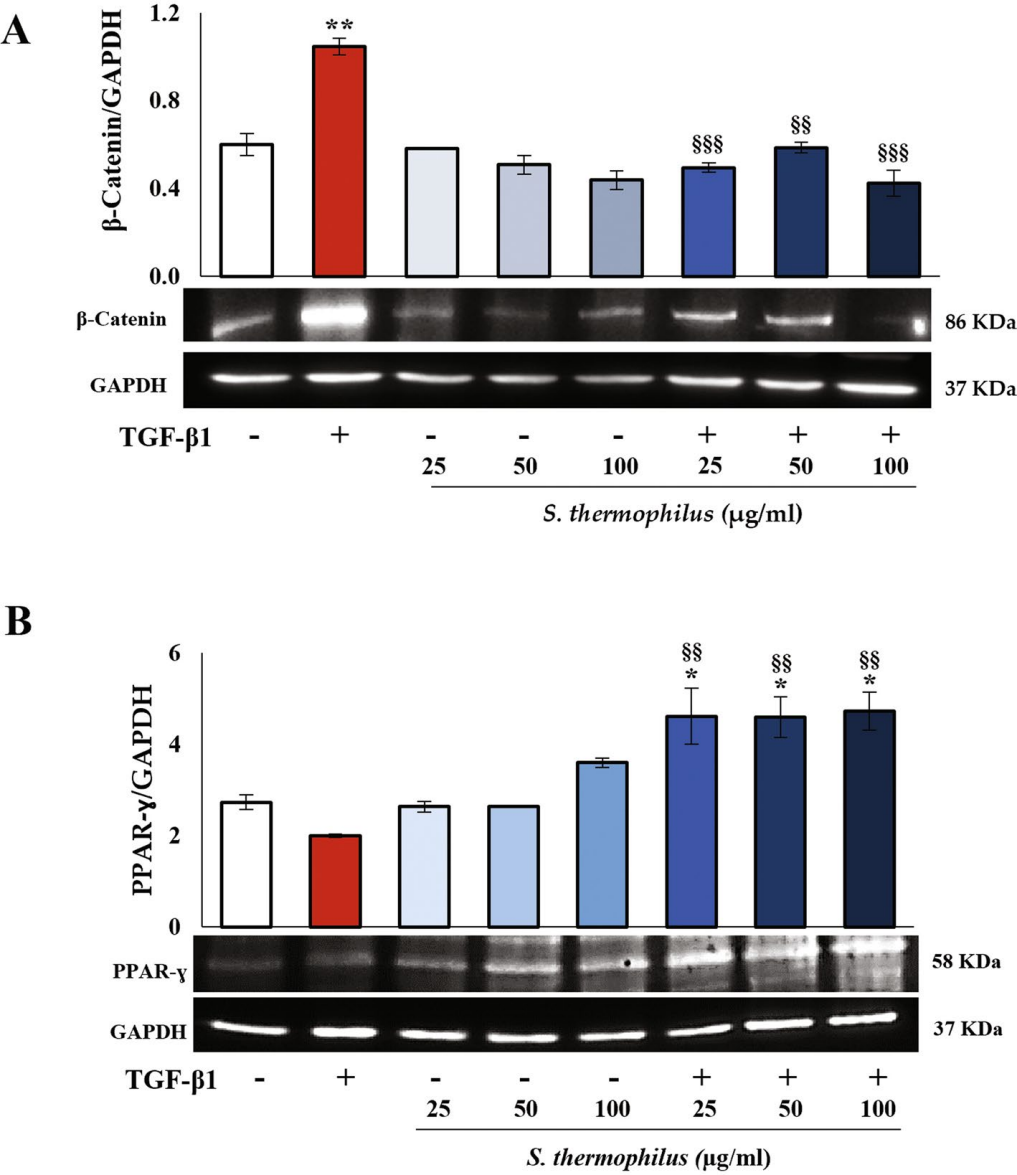
Effect of *S. thermophilus* lysate on β-catenin and PPARγ expression in TGF-β1-activated NHDF.** A** β-catenin and **B** PPARγ protein expressions were evaluated on NHDF incubated as above described, by western blot analysis. Following densitometric analysis, obtained values were normalized vs. GAPDH. Values are expressed as mean ± SEM of two independent experiments in duplicate. For comparative analysis of data, a one-way analysis of variance (ANOVA) with post hoc Tukey test was used (*P < 0.05, **P < 0.01 vs. CNTR; §§ *P* < 0.01, §§§ *P* < 0.001 vs. TGF-β1). Representative images of immunoblotting for β-catenin, PPARγ, and GAPDH are shown

To further assess potential targets of *S. thermophilus* lysate within TGF-β1 signaling pathways, we investigated the effects of bacterial lysate on β-catenin, a key component involved in Wnt/β-catenin signaling whose aberrant activation occurs in the development of skin fibrosis [6]. A significant up-regulation of β-catenin levels was found in TGF-β1-activated NHDF, while no significant difference was registered between the control and *S. thermophilus* lysate-treated cells (Fig. 5A). Notably, *S. thermophilus* lysate treatment abolished the TGF-β1-induced increase of β-catenin levels at all tested concentrations, thus suggesting that the anti-fibrotic activity of the probiotic also involved the inhibition of β-catenin signaling.

Finally, by using western blot assays, we explore whether the β-catenin decrease observed in TGF-β1-treated NHDF after exposure to the probiotic lysate was associated with an increased PPARγ level. Interestingly, an increase of PPARγ was observed following the addition of bacterial lysate to TGF-β1-activated NHDF for 48 h at all concentrations. Although not significantly, PPARγ levels showed a trend to decreased in activated NHDF with respect to the control (Fig. 5B).

## Discussion

Probiotics are well-known to exert beneficial activities against a plethora of pathological conditions including, but not limited to, infectious diseases, diabetes, obesity, inflammatory diseases, cancer, and allergy [33]. In the context of skin disorders, the beneficial effects associated with oral consumption of probiotics have been proven to be effective in promoting healthy skin microbioma composition and in achieving clinically satisfactory results against different clinical conditions such as atopic dermatitis, acne, rosacea, and psoriasis [34, 35]. Moreover, the use of the topical application of probiotics as an effective therapeutic approach for treating patients with dermatological diseases remains undervalued [23]. Even though a few studies have investigated the effects of probiotics on scar formation, probiotics might also play a beneficial role directly in fibrotic tissue associated with dysregulated wound healing, such as hypertrophic scar and keloids. The topical application of *Lactobacillus plantarum* has been reported to be able in reducing the scarring of burn wounds and in mitigating infection, often associated with the development of a hypertrophic scar [36]. Furthermore, a recent study proved that the oral administration of *Lactobacillus rhamnosus* is associated with accelerated wound closure, as well as with the alleviation of scar formation in a murine model of excisional skin wounds [25].

Here, we provide evidence that the exposure to lysate from *S. thermophilus* DSM 24731 is able to counteract the fibrotic process induced in vitro by TGF-β1 by inhibiting the TGF-β1/Smad signaling. Fibroblast migration and proliferation are known to play a crucial role in the pathological healing process and are regulated by numerous factors. Such cellular processes were strongly affected by the presence of the probiotic lysate. Also, the phenoconversion of myofibroblasts was prevented by the exposure to *S. thermophilus* lysate in a dose-dependent manner, as showed by the reduction of α-SMA and fibronectin levels up-regulated by TGF-β1. Furthermore, the bacterial lysate was able to inhibit the capacity of TGF-β1 activated fibroblasts to synthesize Coll I and contract collagen gel. Of note, *S. thermophilus* also abrogated the transcription of the TGF-β1 gene, thus blocking the TGF-β1 induced self-maintaining machinery.

The peculiar fibrogenic actions of TGF-β1 require the co-activation of multiple molecular pathways often interconnected, including the classical (Smad-dependent) or non-classical signaling, among which, the Wnt/β-catenin pathway plays a central role in regulating the dermal fibrosis [6]. As expected, β-catenin levels were significantly increased in TGF-β1-activated NHDF while treatment with *S. thermophilus* was able to reduce them. The activation of Wnt signaling is closely associated with the downregulation of PPARγ, being these two systems able to act in an opposite manner. PPARγ acts in decreasing the fibrotic process by opposing TGF-β1 activity. Numerous agonists of PPARγ minimize fibrotic events such as fibro-proliferation and collage deposition, encouraging the correct repair of skin tissue. The increase of β-catenin by TGF-β1 treatment in NHDF was associated with reduced PPARγ levels. Notably, the probiotic was able to counteract the effect of TGF-β1, reducing β-catenin and increasing PPARγ levels, acting as an anti-fibrotic agent. A limitation of our study is the lack of the identification of the active component/s which individuation will help to deepen further the mechanisms of action behind the observed anti-fibrotic effects of the probiotic lysate. Therefore, the next steps of our research should focus on the important task of identifying and purifying the *S. thermophilus*-derived factor(s), which specifically reverses the skin fibrosis process and affects the involvement of multiple fibrotic pathways. In this context, of particular interest will be to investigate the potential involvement of Toll-like Receptors (TLRs) expressed by fibroblasts able to recognize one or more components from microbes-associated molecular patterns (MAMPs) in the probiotic lysate and the downstream effects in terms of synthesis of cytokines known to influence TGF-β1-induced fibrotic process. Of note, the ability of *S. thermophilus* to activate the heterodimer TLR2/TLR6, which could elicit IL-10 secretion by phorbol myristate acetate-differentiated THP1 macrophages, has been reported [37]. Thus, lactic acid bacteria strains, including *S. thermophilus*, stimulating TLR2/TLR6 pathways, might modulate excessive inflammatory reactions. It could be crucial to demonstrate that one or more components of MAMPs from *S. thermophilus* lysate can interact with the TLR2/TLR6 heterodimer by inducing the release of IL-10 by fibroblasts. This could explain, at least in part, the anti-fibrotic effect exerted by the used probiotic strain. In fact, IL-10 is considered a potent anti-inflammatory cytokine which plays a pivotal role in wound healing and scar formation, exerting inhibitory effects on the excessive deposition of extracellular matrix components and fibroblast-to-myofibroblast transition induced by TGF-β1 [38, 39].

## Conclusion

To the best of our knowledge, the results of the present study provide the first evidence of the ability of *S. thermophilus* to counteract the pro-fibrotic effects induced by TGF-β1 in a fibrosis skin model. In the current scenario, the available therapeutic strategies may have variable success rates or weak responses with recurrences after treatment. Moreover, the considerable side effects that follow the treatment cause discontinuation of the therapy. Therefore, the medical need is directed toward well- tolerated therapies able to prevent or reverse fibrosis without side effects as far as possible. In light of the encouraging results obtained in vitro, our next goal is to plan a prospective study in collaboration with clinicians to investigate the effect topical probiotic lysate on surgical wounds in patients with skin healing disorders. At the same time, we will be also interested to study the effects of topical application of *S. thermophilus* lysate on hypertrophic scars and keloids by selecting appropriate probiotic lysate delivering system.

## Materials and methods

### Preparation of bacterial samples for cell treatments

*Streptococcus thermophilus* DSM 24731® in a pure lyophilized form was kindly provided by Prof. Claudio De Simone, MD. For bacterial fraction preparation, stocks of 600 × 10^9^ CFU/g of lyophilized probiotic resuspended in Phosphate Buffered Saline (PBS, Euro Clone, West York, UK) were centrifuged at 8600 ×g, washed twice, resuspended in 10 ml of PBS and sonicated (30 min, alternating 10 s of sonication and 10 s of pause) using a Vibracell sonicator (Sonic and Materials, Danbury, CT, USA). The sample’s absorbance was evaluated at 590 nm to verify bacterial cell disruption before and after every sonication step (Eppendorf Hamburg, Germany). The samples were then centrifuged at 17,949 × g and the supernatants filtered using a 0.22-µm-pore filter (Corning Incorporated, Corning, NY, USA) to remove any whole bacteria remaining and obtain the bacterial lysate. DC Protein Assay (BioRad, Hercules, CA, USA) was performed using bovine serum albumin as the standard, to determine the total protein content.

For cell treatments, bacterial lysate was prepared to obtain final concentrations of 25, 50, or 100 µg protein/ml, equivalent to 1.87 × 10^8^ CFU/ml, 3.75 × 10^8^ CFU/ml and 7.5 × 10^8^ CFU/ml, respectively.

### Cell line and culture conditions

Normal adult primary human dermal fibroblast (NHDF, Adult CC-2511) cell line, was purchased from Lonza/Cell Applications, (Basel, Switzerland) and then cultured at low passage (*N* = 6–9) in DMEM supplemented with 10% of fetal bovine serum (FBS), 100 U/ml penicillin, 100 mg/ml streptomycin, and 2 mM glutamine. The cells were maintained in a humidified atmosphere of 95% air and 5% CO_2_ at 37 °C. The reagents for cell biology and consumables were obtained from EuroClone (EuroClone, West York, UK), if not otherwise declared. After reaching 80% confluence, the cells were sub-cultured; the experiments were carried out at the 15^th^ passage.

### Cell treatment and analysis of cell growth and viability

Analysis of cell growth was performed utilizing the IncuCyte® Live Cell Imager system (Essen BioSciences, Inc., Ann Arbor, MI, USA) for real time evaluation of cell confluence. Briefly, cells were plated in a 96-multiwell culture plate at 2.5 × 10^3^ cells/cm^2^, allowed to attach overnight and starved for 24 h in DMEM containing 0.5% FBS. After, the cells were incubated with TGF-β1 (10 ng/ml), or lysate from *S. thermophilus* (25, 50, or 100 µg protein/ml) or with the relative combinations in DMEM with 0.5% FBS. Culture plates were sited into the IncuCyte® Live Cell imager, and images were captured using phase contrast channel and were taken every 4 h from 0 to 72 h post treatment. Two image sets were acquired from several points of the well, using a 10X objective lens, and all the treatment conditions were run in triplicates. At the end of the experiment, the percentage of cell confluence was measured and analyzed by IncuCyte ZOOM™ software (Essen BioSciences, Inc.) and was used to indicate the proliferation rate.

For all of the other experiments, NHDF were plated at a density of 8000 cells/cm^2^, grown overnight, and starved for 24 h in DMEM containing 0.5% FBS. This was followed by a stimulation for 48 h with human transforming growth factor (hTGF-β1; Cell signaling Technology, MA, USA) (10 ng/ml) to induce the fibrotic phenotype. Stimulation was performed both in the presence and absence of bacterial fraction from *S. thermophilus* at different final concentrations (25, 50, or 100 µg protein/ml) in order to observe its effects. After treatments, the cells were collected and centrifuged for 10 min at 400 × g. To analyze their number and viability, pellets were incubated with 0.04% trypan blue (Euro Clone, West York, UK) for 5 min, untreated cells were used as controls. Cells were then transferred to a Bürker chamber and counted by microscopy (Eclipse 50i, Nikon Corporation, Tokyo, Japan).

### Scratch Wound Healing assays

To evaluate the effect of *S. thermophilus* lysate at different concentrations on TGF-β1-induced migration in the NHDF, a scratch assay was performed as previously described [40]. Briefly, the cells were plated at 20 × 10^4^/cm^2^ in multiwell plates and cultured until reaching confluence. DMEM was removed and the cell monolayers were scratched using a 200 µl pipet tip. Subsequently, the cells were washed with PBS and then treated with cultured with TGF-β1 (10 ng/ml), in presence or absence of bacterial lysate (25, 50, or 100 µg protein/ml). The images of cell migration were taken by the inverted light microscope (Eclipse TS 100, Nikon) at 10X magnification at different time points after the injury (0–48 h). The experiments were conducted in duplicate, and six fields for were analyzed for each condition. The images were analyzed quantitatively while using the standalone TScratch software that automatically calculates the portion of area that is occupied by the cells by a mathematical model to calculate the percentage of wound closure.

### Western blot analysis

For protein analysis through western blot, cell pellets previously collected were washed in PBS and then lysed in RIPA buffer (Merck KGaA, Darmstadt, Germany) containing a protease inhibitor mixture (5 µg/ml carboxypeptidase inhibitor, 5 µg/ml trypsin inhibitor, 1 mM PMSF, 10 µg/ml leupeptin, 10 µg/ml aprotinin, and 10 µg/ml pepstatin) (Sigma Aldrich, St. Louis, MO, USA). Protein content of samples was then measured with DC Protein Assay (BioRad, Hercules, CA, USA) using BSA as standard. Samples were then prepared for western blot loading by mixing 25 µg of proteins with sample buffer, followed by a 5 min. boiling at 100 °C. Proteins were then separated by 10% SDS-polyacrylamide gel electrophoresis. Transfer of proteins was performed onto a 0.45 μm nitrocellulose membrane sheet (BioRad), for 1 h at 4 °C at 70 V, using a Mini Trans-Blot Cell apparatus (BioRad). Blocking of membranes was done by soaking the membrane in 5% non-fat dry milk for 1 h at room temperature. Overnight incubation was done at 4 °C with the following antibodies: rabbit monoclonal antibody anti-human phospho-Smad2/3 (phospho-S465/S467, clone 1074 A), (R&D Systems Inc., Minneapolis, MN, USA) 1:100, or with mouse monoclonal antibody anti-α-actin smooth muscle (ACTA2, α-SMA, clone 4A4) (OriGene, Rockville, MA, USA) 1:1000, or with rabbit monoclonal anti-Fibronectin/FN1, (clone E5H6X) (Cell Signaling Technology, Danvers, MA, USA) 1:1000 or with rabbit polyclonal antibody anti-COL1A1, (catalogue number PA 2140-2) (Boster Biological Technology, Pleasanton, CA, USA) 1:1000, or with rabbit polyclonal antibody anti-β-catenin (catalogue number 9581) (Cell Signaling Technology, Danvers, MA, USA) 1:1000, or with rabbit monoclonal antibody anti-PPARγ (clone C26H12) (Cell Signaling Technology, Danvers, MA, USA) 1:1000, and mouse monoclonal antibody anti-GAPDH (clone OTI2D9) (OriGene, Rockville, MA, USA) 1:1000. Horseradish peroxidase (HRP)-conjugated goat anti-rabbit IgG secondary antibody at 1:2000 was used for anti-human phospho-Smad2/3, anti-Fibronectin, anti-COL1A1, anti-β-catenin and anti-PPARγ antibodies, and horseradish peroxidase (HRP)-conjugated rabbit anti-mouse IgG secondary antibody at 1:2000 for anti-α-SMA and anti-GAPDH antibodies (Millipore EMD, Darmstadt, Germany). Immuno-reactive protein bands were visualized by enhanced chemiluminescence (ECL, Amersham Pharmacia Biotech), according to the manufacturer’s instructions. Band densities were determined using ALLIANCE (UVITEC, Cambridge UK) chemiluminescence documentation system and normalized to the relative GAPDH bands. Values were given as relative units.

### Immunofluorescence staining for fibrotic markers

NHDF were grown on coverslips in a 12-well plate (seeded at 8 × 10^3^ cells/cm^2^) and treated as specified above for the indicated time points. At the end of treatment, the coverslips were washed with PBS, fixed with 4% formaldehyde for 20 min., permeabilized with 0.1% Triton X-100 (Sigma-Aldrich, St. Louis, MO, USA) for 5 min., and blocked with 3% BSA (Sigma Aldrich, St. Louis, MO, USA) for 20 min, at room temperature. Coverslips were then incubated overnight at 4 °C with mouse monoclonal antibody anti-α-actin smooth muscle (ACTA2, α-SMA) (OriGene, Rockville, MA, USA) 1:250, or rabbit monoclonal anti-fibronectin/FN1, (Cell Signaling Technology, Danvers, MA, USA) 1:200, or rabbit polyclonal antibody anti-COL1A1 (Boster Biological Technology, Pleasanton, CA, USA) 1:250. Subsequently, staining was performed using a FITC conjugated goat anti-rabbit polyclonal IgG secondary antibody (Millipore EMD, Darmstadt, Germany) 1:1000 or FITC conjugated goat anti-mouse polyclonal IgG secondary antibody (Bethyl Laboratories, Inc, Montgomery, TX, USA) for 1 h at room temperature. All samples were then incubated with TRITC labeled phalloidin (Sigma-Aldrich) for 45 min at room temperature. The coverslips were mounted with VECTASHIELD® Antifade Mounting Medium with DAPI (Vector Laboratories, Inc., Burlingame, CA, USA) and examined at 100X magnifications with fluorescence microscopy (Eclipse 50i, Nikon, Tokyo, Japan).

### Collagen gel retraction assay

To assess the contraction capacity of NHDF, the cells were seeded into three-dimensional collagen lattices based on a previously described technique [41]. Briefly, the acid-extracted type I collagen (5 mg/ml) mixture was prepared on ice with rat tail collagen I (Enzo Life Sciences, Lausen, Switzerland) in acetic acid 0.2% and then diluted at 3 mg/mL in filter-sterilized water. NHDF were resuspended in complete media at 8 × 10^5^ cells/ml.

Resuspended cells (8 × 10^4^/100 µl) were added to 300 µl complete media, 200 µl collagen mixture (3 mg/ml) and brought to neutral pH with NaOH on ice. 500 µl of the collagen/cell mixture were added to each well of a pre-warmed 12-well non-tissue culture treated plate (Corning Incorporated, Corning, NY, USA). Gels polymerized at 37 °C for 1 h and covered with media containing or not 10 ng/ml TGF-β1, *S. thermophilus* lysate (25, 50, or 100 µg protein/ml) and their combination. After the addition of treatments, the gels were carefully detached from the well by gently running the tip along the gel edges. Lattices were imaged with a digital camera at a fixed distance above gels, to obtain an image before release and at multiple times after release. Lattice area was measured with ImageJ and normalized to pre-release area (T0).

### Total RNA extraction and quantitative real-time PCR (qPCR)

Gene expression analysis of TGF-β1 was performed in untreated (CNTR) and treated NHDF as for protein expression experiments, via real-time RT-PCR using a ViiA7 sequence detection system (Applied Biosystems, Foster City, CA, USA). Total RNA was extracted from cells using the RNeasy Mini Kit (QIAGEN, Hilden, Germany) according to the manufacturer’s instructions and quantified by spectrophotometry. An amount of 1$\mu$g of total RNA was reverse transcribed in a final volume of 20 µL using a mixture of random primers below reported. An amount of 0.5 µg of each cDNA was used to perform the real-time PCR. Real-time quantitative RT-PCR analysis was carried out by SYBR Green dye detection (Thermo Fisher Scientific Waltham, MA, USA) according to the manufacturer’s instructions. Reverse and forward primers, purchased from IDT (acquired from Integrated DNA Technologies, IDT, Coralville, IA, USA), were used at a concentration of 1 µM (hTGF-β1), and their sequences were as follows: hTGF-β1 forward 50-CAACGAAATCTATGACAAGTTCAAGCAG-30 and reverse 50- CTTCTCGGAGCTCTGATGTG-30. In NHDF, as an internal control for gene expression normalization, the mRNA level of GAPDH was measured by following primers: GAPDH forward 50-TTGCCCTCAACGACCACTTT-30 and reverse 50- TGGTCCAGGGGTCTTACTCC-30. The fold-change quantification of target genes was calculated with the 2^− DDCt^ method. The samples were run in triplicate, and the experiment was repeated twice.

### Statistical analysis

The data were analyzed using GraphPad Prism version 6.01, (GraphPad Software, San Diego, CA, USA). A one-way ANOVA or a two-way ANOVA, followed by Tukey post hoc test, was used to compare the mean values among groups. Results are expressed as mean ± SD or mean ± SEM of three independent experiments performed in triplicate. In each case *P* values ≤ 0.05 were considered statistically significant.

## Supplementary Information

**Additional file 1.**

## Data Availability

The datasets used and/or analyzed during the current study are available from the corresponding author on reasonable request.

## Abbreviations

NHDF: Normal human dermal fibroblasts
TGF-β1: Transforming Growth Factor-βeta1
α-SMA: Alpha-Smooth muscle actin
Smad: Small mothers against decapentaplegic
PPARγ: Peroxisome proliferator-activated receptor gamma
ECM: Extracellular matrix
Wnt: Wingless/Int
ATCC: American Type Culture Collection
qRT‐PCR: Real‐Time Quantitative Reverse Transcription‐Polymerase Chain Reaction
