# Supplementary material for: Efficacy of probiotic Streptococcus thermophilus in counteracting TGF-β1-induced fibrotic response in normal human dermal fibroblasts
Source: J Inflamm (Lond). 2022 Dec 19;19:27. doi: 10.1186/s12950-022-00324-9 (PMC9764521; doi:10.1186/s12950-022-00324-9)

Supplementary materials

Efficacy of probiotic *Streptococcus thermophilus* in counteracting TGF-β1-induced fibrotic response in normal human dermal fibroblasts

Francesca Lombardi, Francesca Rosaria Augello, Serena Artone, Blerina Bahiti, Jenna Marie Sheldon, Maurizio Giuliani, Maria Grazia Cifone, Paola Palumbo, Benedetta Cinque

Fig. 2A

α-SMA

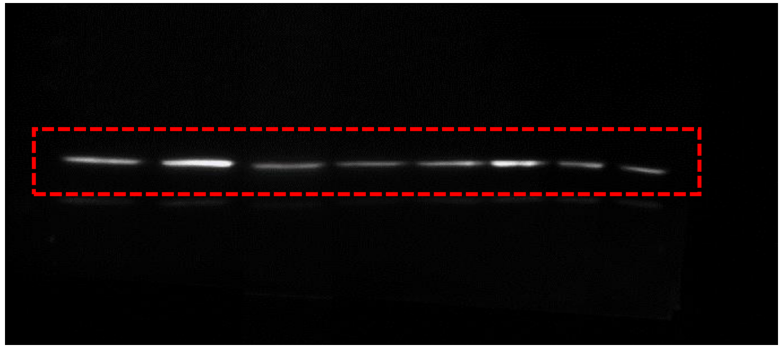

42 KDa

GAPDH

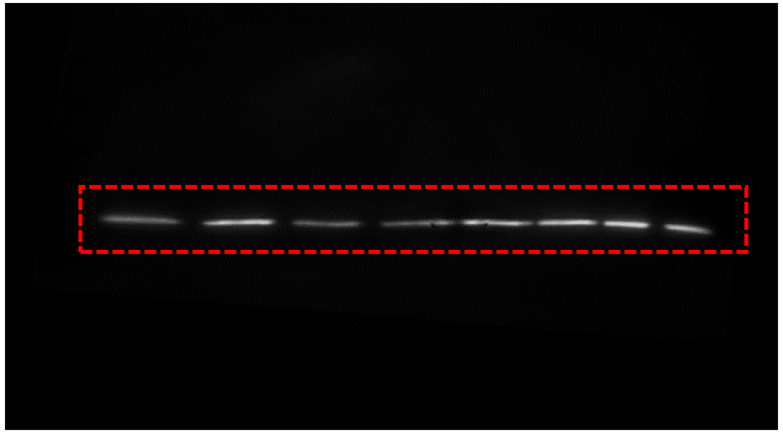

37 KDa

Fig. 2C

Fibronectin

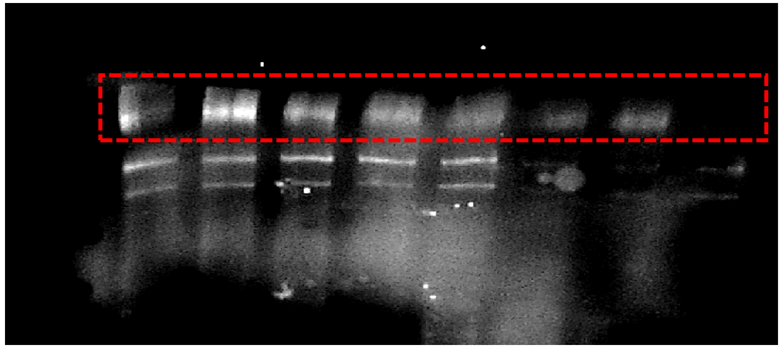

300 KDa

GAPDH

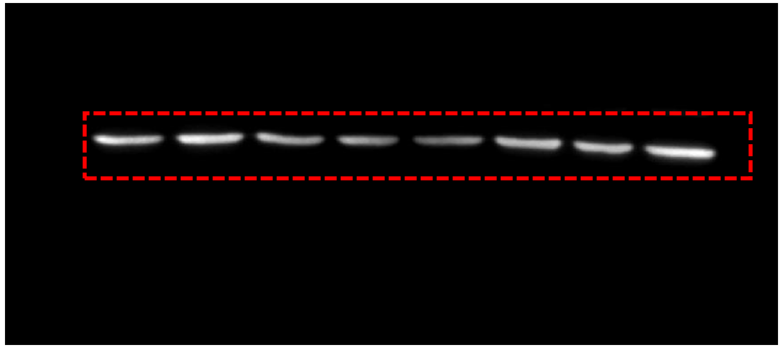

37 KDa

Fig. 3

Collagen I

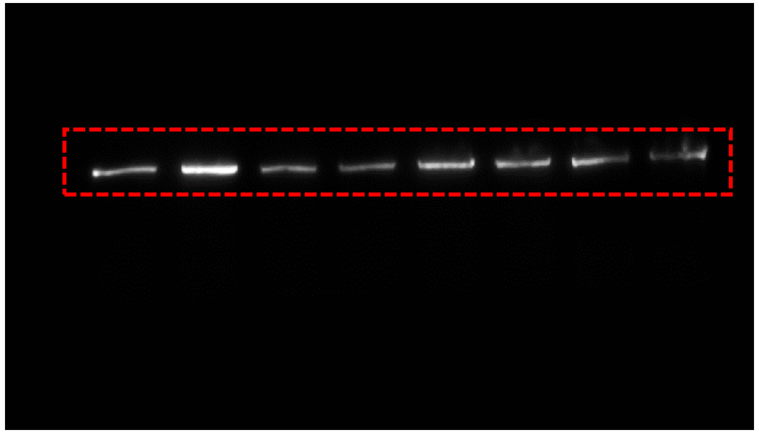

130 KDa

GAPDH

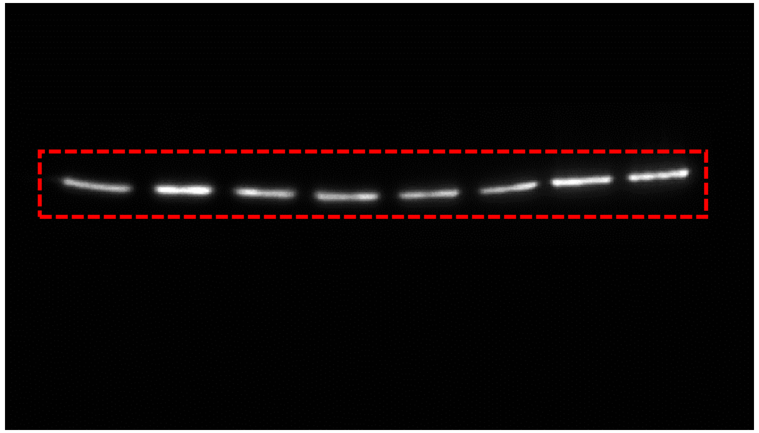

37 KDa

Fig. 4A

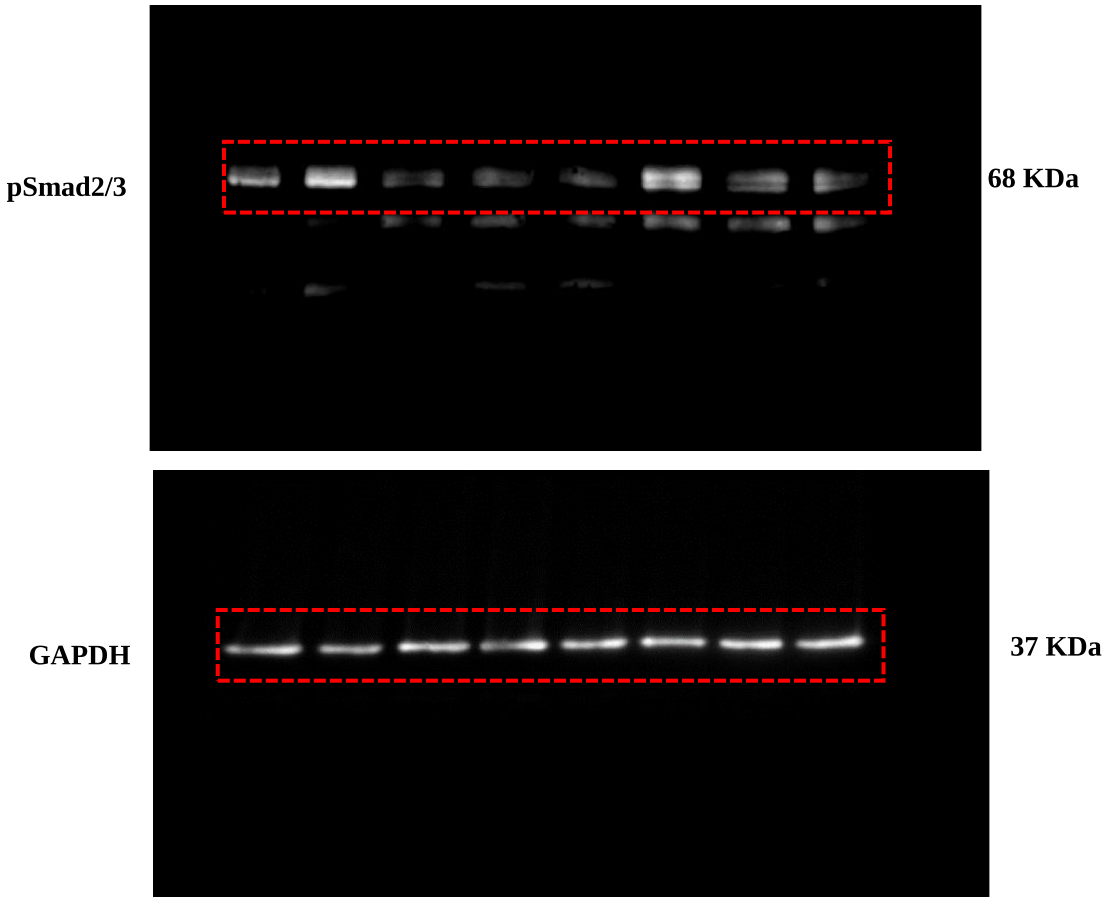

Fig. 5A

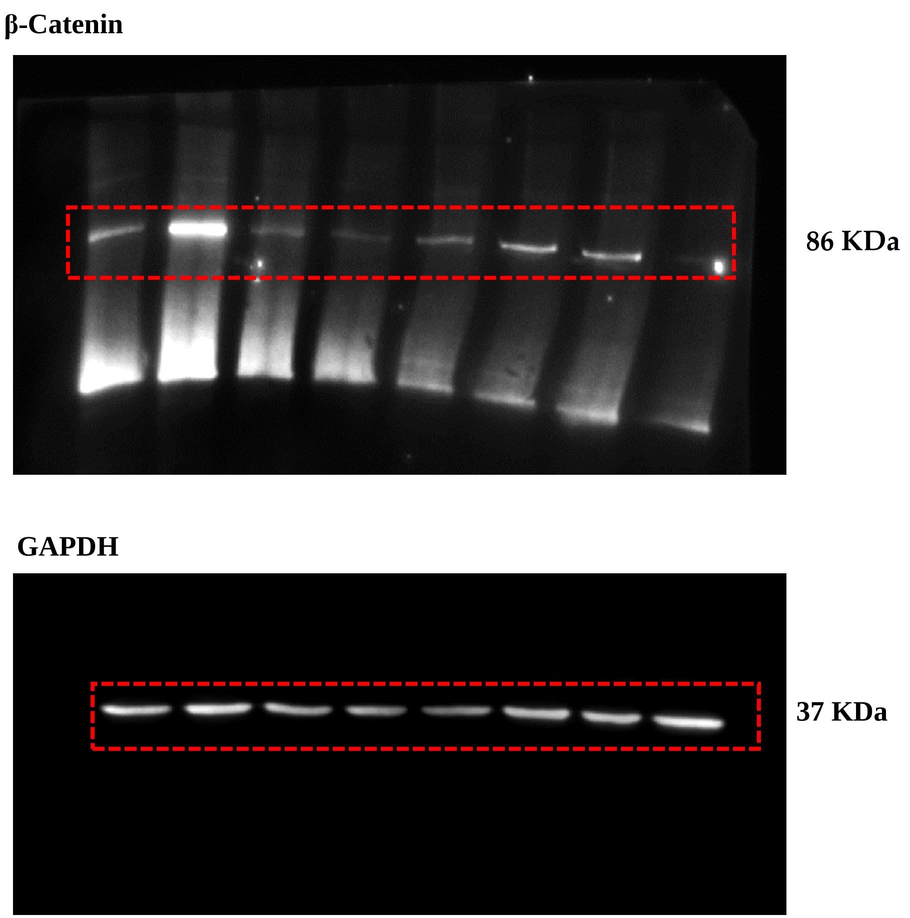

Fig. 5B

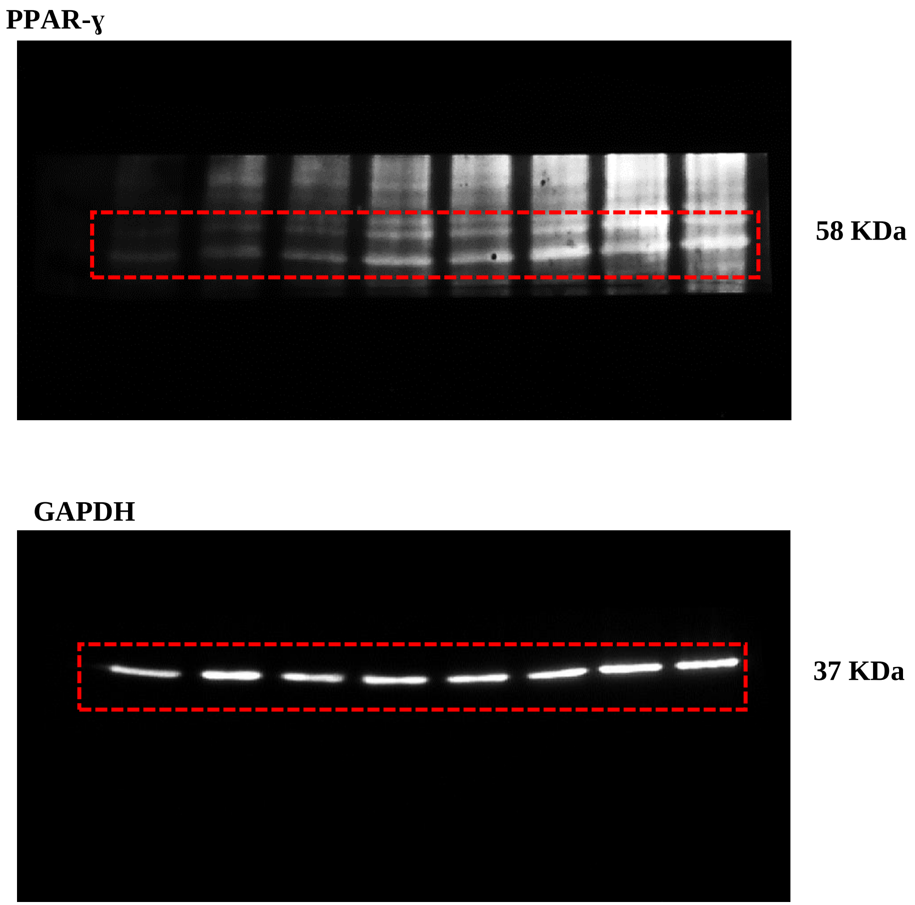

Supplement: Supplementary file 1 — Additional file 1. [file 12950_2022_324_MOESM1_ESM.pdf]
